# Pollination and fruit infestation under artificial light at night:light colour matters

**DOI:** 10.1038/s41598-020-75471-1

**Published:** 2020-10-27

**Authors:** Michiel P. Boom, Kamiel Spoelstra, Arjen Biere, Eva Knop, Marcel E. Visser

**Affiliations:** 1 Department of Animal Ecology, Netherlands Institute of Ecology (NIOO-KNAW), P.O. Box 50, 6700 AB Wageningen, The Netherlands; 2 Department of Terrestrial Ecology, Netherlands Institute of Ecology (NIOO-KNAW), P.O. Box 50, 6700 AB Wageningen, The Netherlands; 3 Department of Evoluationary Biology and Environmental Studies, University of Zürich, Winterthurerstr. 190, 8057 Zürich, Switzerland; 4 Agroscope, Agroecology and Environment, Reckenholzstr. 191, 8046 Zürich, Switzerland

**Keywords:** Behavioural ecology, Environmental impact

## Abstract

Rapid human population growth and associated urbanization lead to increased artificial illumination of the environment. By changing the natural light–dark cycle, artificial lighting can affect the functioning of natural ecosystems. Many plants rely on insects in order to reproduce but these insects are known to be disturbed by artificial light. Therefore, plant–insect interactions may be affected when exposed to artificial illumination. These effects can potentially be reduced by using different light spectra than white light. We studied the effect of artificial lighting on plant–insect interactions in the *Silene latifolia*–*Hadena bicruris* system using a field set-up with four different light treatments: red, green, white and a dark control. We compared the proportion of fertilized flowers and fertilized ovules as well as the infestation of fruits by *Hadena bicruris,* a pollinating seed predator. We found no difference in the proportion of fertilized flowers among the treatments. The proportion of fruits infested by *H. bicruris* was however significantly higher under green and white light and a significantly lower proportion of fertilized ovules was found under green light. We show that artificial light with different colours impacts plant–insect interactions differently, with direct consequences for plant fitness.

## Introduction

Continuous human population growth and related urbanisation cause an increase in artificially illuminated areas by approximately 2.2% during recent years^1^. The worldwide increasing light pollution causes a distortion of natural light–dark cycles, with consequences for the survival and reproduction of organisms and the timing of biological activities^2^. For example, it has been suggested that artificial light at night can have important consequences for plants that have adjusted to natural light–dark cycles^3^. Effects of light at night may as well cascade between trophic levels, for example by changes in herbivory^4,5^, which can be both resource and predation controlled^6^. Also, light at night can interfere with the activity and behaviour of nocturnal moths^7^ and disrupt plant-pollinator interactions with negative consequences for pollination^8^. This might be detrimental for plants as many plant species are dependent on pollinators for their reproduction^9,10^ and several nocturnal moths are considered to play a vital role as pollinators^11–13^.

One way plant-pollinator interactions might be disrupted is distinct flight to light behaviour of many nocturnal insects^7,14,15^, which can distract them from visiting the flowers. This attraction to artificial light is also an important cause of mortality among flying insects^16,17^ and can lead to reduction of moth population numbers^18^. Furthermore, artificial light at night might affect nocturnal pollinators and the pollination service they provide by altering their feeding and mating behaviour^19,20^. Artificial light can disrupt flight to mating and feeding locations^17,21^, and can distort the male response to pheromone as well as supress pheromone release of females^22–24^.

Despite an increased interest in the effects of artificial illumination on nature, little attention has been given so far to different impacts of various colours of light. Although it is well known that nocturnal insects are attracted to light, important differences exist related to spectral composition. Donners et al*.*^25^ show, by means of a theoretical model of the insect eye, that nocturnal insects in general are mostly sensitive to UV-light and shorter wavelengths. Van Langevelde et al*.*^14^ showed the abundance and species richness of moths around artificial light sources differs depending on the wavelength, with in general higher abundance and species richness occurring with shorter wavelengths (381.8 nm; violet light). Although in the study of van Grunsven et al.^18^ there was no difference between white, green and red light in the decline of moth numbers relative to the dark control. How this relation with wavelength might cascade to important ecosystem function of moths, such as pollination, remains largely unclear (but see Knop et al*.*^8^).

In this study we investigated the impact of different colours of artificial light on the plant insect interaction using the *Silene latifolia*–*Hadena bicruris* study system. *Silene latifolia* is a dioecious plant, with the nursery pollinator *H. bicruris* being night-active and considered to be the most important pollinator^26^. While females visit more flowers, both males and females of *H. bicruris* act as pollinators, showing no preference for flowers of one sex^27^*.* When female *H. bicruris* pollinate *S. latifolia* flowers, they can additionally lay eggs on the ovary and the offspring consequently consumes the developing seeds^28^. Thus, *H. bicruris* is both a pollinator and a herbivore for *S. latifolia*. It recently has been shown that the number of laid eggs in the flowers of *S. latifolia* is increased in areas adjacent to artificially illuminated areas but not in areas directly illuminated, most likely due to the fact that females of *H. bicruris* are able to fly away from artificial light sources and prefer dark oviposition sites^21^. Pollination of *S. latifolia* on the other hand seems not to be affected by light^21^. The study, however, focused on white light only, and we currently do not know whether the effect of light on the *S. latifolia*–*H. bicruris* relationship vary depending on the light colour.

We thus used a long term artificial lighting project, Light on Nature^29^, as an experimental set up. We used four different natural areas which were illuminated with three different colours of artificial light (white, green and red) and a dark control. As different colours of artificial light are known to attract pollinator species differently^14^, the number of potential pollinators in the close vicinity of the lamp might vary depending on the colour of the light. Furthermore, pollinators which are not fatally attracted to the lamp (i.e. circulate around the lamp until exhaustion) might be increased in density around the light source, but continue visiting flowers thereby increasing the proportion of flowers fertilized in illuminated areas compared to dark controls. Alternatively, they could be distracted by the light as shown by Knop et al*.*^8^ reducing the proportion of flowers fertilized in illuminated areas compared to dark controls. Attraction to specific light colours might also invoke a difference in abundance among *H. bicruris* resulting in consequences for pollination, infestation rates and related seed predation. Alternatively, *H. bicruris* might avoid illuminated flowers for oviposition, thereby increasing infestation rates and seed predation in dark areas adjacent to light sources^21^.

## Materials and methods

### Experimental setup

For this research, we made use of four field sites in the Netherlands (Lebretshoeve, Radio Kootwijk and Klaterweg 1 & 2). These sites are part of a long-term illumination experiment: Light on Nature (LON), and have been illuminated since early 2012. For details on the experimental set-up of the Light on Nature project see Spoelstra et al*.*^29^. Within this set up, three different light colours (red, green and white—see Spoelstra et al.^29^ and Fig. S1 for the spectra) and a dark control (lamppost without light) are used in previously not illuminated nature areas (see Fig. S2 for an impression of the set up). All spectra are broad and continuous, and were selected for suitability for public lighting because they allow for full colour vision in humans^29^. The green spectrum contains more blue and less red light and was originally designed to minimize impact on migrating birds. The red spectrum has less blue and more red light and was designed to minimize impact on nocturnal animals^29,30^. Every research site has four 100 m long transects, placed perpendicular in the forest edge with an average distance of 207 ± 17 m (mean ± s.e.) between transects. At each transect one of the four treatments is delivered (three spectra and dark control, randomly assigned to transects within each site) by five 4 m tall light posts placed 25 m apart. The intensity of the three spectra was normalized to Lux, with an average of 7.6 ± 1.2 lx at ground level. The different research sites all represent different forest types. *S. latifolia* is a native species within our research area^31^, although it does not grow at the research sites as a result of unfavourable soil properties.

Plants were grown in arch-greenhouses before placing them in the field to ensure similar growing conditions and development. We selected plants for placement in the field based on the timing of flowering and physical condition of the plant (plants needed to be strong enough to endure the transport to the field). Plants were placed outside when they were at the verge of flowering to make sure that most flowers were produced during the period in the field. Eight plants were placed in a crate (± 20 cm apart) with a layer of sand for stability and a layer of water to prevent dehydration. We placed three crates at each transect, resulting in a total of 24 plants per transect. We aimed at placing twice as many female plants, so approximately 16 females and 8 males were used at each transect. This deviated slightly due to the availability of female and male plants and the accuracy of the sex-determination of the plants when they were not yet flowering. Overall, male:female ratio ranged between 6:18 and 11:13. We placed the plants directly beneath the lamppost at the forest edge. The location at the forest edge was selected to meet the light requirements of the plant species^32^. Plants were left in the field for 13–14 days and were regularly checked and watered when necessary.

After 13–14 days the number of produced flowers was counted based on the number of pedicels that remained when the flower was not fertilized, and the number of fruits present when fertilization took place (fruit set: flowers producing fruit/all flowers). The number of fertilized flowers represents a lower estimate of the number of pollinated flowers as the number of remaining pedicels may include some early aborted fruits. Every fruit was checked for infestation by *H. bicruris* by removing the sepals and looking for eggs, caterpillars, or holes in the seed capsule^33^. By doing this we were able to determine the proportion of fruits infested (fruit predation: fruits infested/flowers producing fruits) by *H. bicruris.*

To look at pollination effectivity, we sampled two uninfested fruits from each plant when possible (some plants did not have uninfested fruits). The sampled fruits were opened and a scan of the content was made. The scans were analysed with the program WinSeedle Pro^34^. Based on ellipsoid surface area, we distinguished between fertilized and unfertilized ovules. Ovules with a surface area smaller than 0.6 mm^2^ were considered unfertilized, while ovules with a surface area between 0.6 and 3 mm^2^ were considered fertilized (based on the two normal distributions observed in the data; Fig. S3). We calculated the proportion of fertilized ovules (seed set) by dividing the larger ovules (> 0.6 mm^2^) through the total of ovules (all ovules between 0 and 3 mm^2^) in each fruit. Note that seed set only refers to a proportion and that fruits might differ in the total number of ovules they produce.

To estimate the potential effect of different light colours on the pollinator’s contribution to variation in female reproductive output, we calculated the per flower reproductive output for each treatment (for every treatment: seed output per flower = (proportion of flowers producing fruit) × (1 − proportion of fruits infested) × (proportion of ovules fertilized) × (average number of ovules per fruit). The corresponding standard errors are calculated using standard error propagation for multiplication.

### Statistical analyses

We analysed all data using R project for statistical computing^35^. Our response variables consisted of proportional data (proportion of flowers fertilized, proportion of fruits infested and proportion of fertilized ovules). We used generalized linear mixed-effect models in the package lme4^36^ in which family (distribution) was specified as “Binomial”. In each GLMM, *study site* was fitted as a random factor while *treatment* (Light) was fitted as a fixed factor. In the analysis of the proportion of fertilized ovules, *fruit* nested in *plant* was also included as a random factor. For post-hoc testing we used Tukey tests in the emmeans package^37^. We tested for differences in estimated per flower reproductive output by using a one-way ANOVA.

## Results

**Figure 1:**
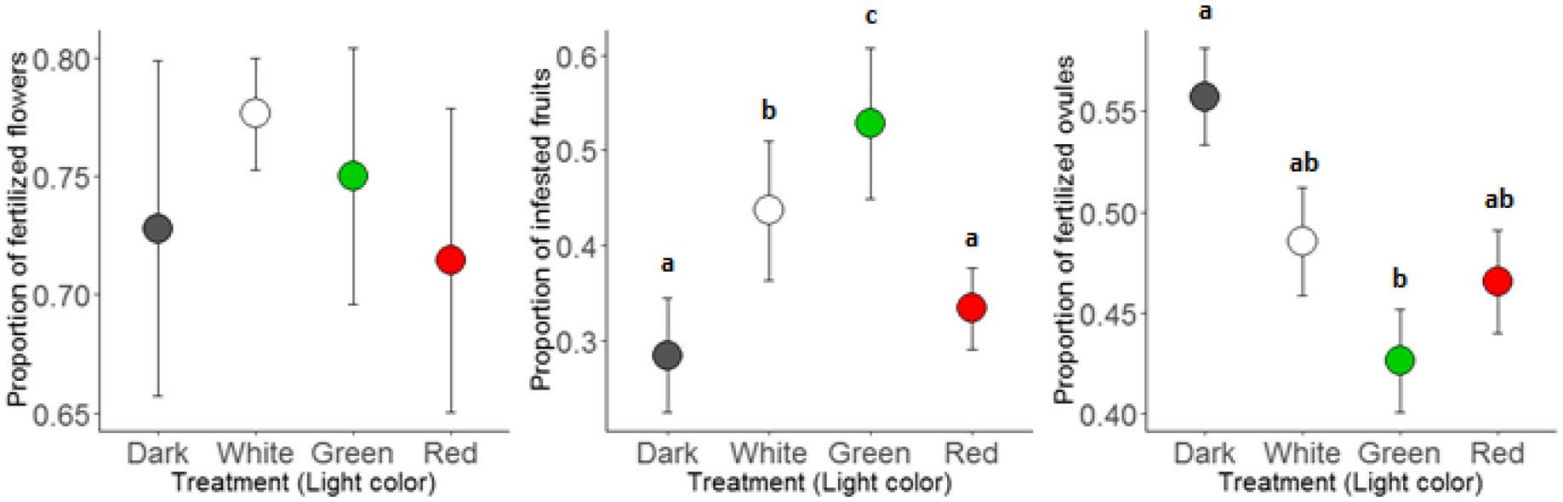
(**a**) Proportion of flowers fertilized per treatment (means ± SE). (**b**) Proportion of fruits infested by *H. bicruris* per treatment (means ± SE). (**c**) Proportion of fertilized ovules (means ± SE). Significant differences (p < 0.05) between treatments are indicated by letters.

We found no significant effect of light treatment on the proportion of fertilized flowers (χ^2^ = 5.06; df = 3; p = 0.17; Fig. 1a), correcting for random effects of the study site. Fertilization proportion ranged between 0.71 (red light) and 0.78 (white light). Light treatment did have a significant effect of the proportion of fruits that was infested by *H. bicruris* (χ^2^ = 81.17; df = 3; p < 0.001). The Tukey post-hoc tests revealed significantly higher infestation rates under white and green light compared to red light and the dark control (Fig. 1b). The highest proportion of infestation was observed under the green light, with an infested proportion of 0.53 compared to 0.44, 0.33 and 0.28 for white light, red light and dark respectively. Differences in the proportions of fertilized ovules were also found to be significant (χ^2^ = 12.96; df = 3; p < 0.01), with green light showing a significantly lower proportion of fertilized ovules than the dark control (Fig. 1c).

Based on the proportions found, we calculated (see “Materials and methods”) the per flower reproductive output (with s.e.) under different light treatments (Table 1). The average number of ovules was found to be 404 ± 7.78 per fruit. Differences between seeds produced per flower under green, white, red light and the dark control were not significantly different (F_3,12_ = 0.60; p = 0.63).

**Table 1:** Proportions of flowers fertilized, fruits infested and ovules fertilized per treatment (mean ± SE), as well as the estimated reproductive output as number of developed seeds per flower.

Treatment	Proportion of flowers fertilized	Proportion of fruits infested	Proportion of ovules fertilized	Estimated reproductive output (number of seeds per flower)
Dark	0.73 ± 0.071 (689)	0.28 ± 0.060 (497)	0.56 ± 0.024 (28,348)	117 ± 40
White	0.78 ± 0.024 (680)	0.44 ± 0.073 (518)	0.49 ± 0.027 (23,822)	86 ± 28
Green	0.75 ± 0.054 (596)	0.53 ± 0.079 (429)	0.43 ± 0.026 (29,127)	61 ± 20
Red	0.71 ± 0.064 (663)	0.33 ± 0.043 (514)	0.47 ± 0.025 (27,650)	90 ± 28

The respective sample sizes are given between brackets for each treatment. Note that for the proportion of flowers fertilized and the proportion of fruits infested the sample size do not refer to independent data points and therefore in the statistical analysis the number of flowers/fruits is nested in transect, which is nested in study site. Similarly, for the proportion of ovules fertilized, the sample size refers to the number of ovules nested in fruit, nested in plant, nested in transect, nested in study site. The estimated reproductive output per flower is calculated based on the average number of ovules per fruit (404 ± 7.78) and the respective proportions of flower fertilization, fruit infestation and ovule fertilization.

## Discussion

We found that under artificial light at night there was no effect of light colour on the proportion of fertilized flowers (fruit set) but that there was a higher proportion of infestation (fruit predation) under green and white light. We put forward two hypotheses to explain these, at first sight, paradoxical results.

These results may be explained from the way light is affecting the behaviour of *H. bicruris,* which is not only a fruit predator but also the main pollinator. Females of *H. bicruris* show no fatal attraction to light at short distances^21^ and hence it may be attracted to these light sources from longer distances, but at closer distances ‘escapes’ from this attraction. Thus, after being attracted over longer distances and leading to a higher density close to the lamp, there were more females to infest the flowers resulting in higher infestation rates under white and green light compared to the dark control. This is in contrast to findings of Giavi et al.^21^, where *H. bicruris* seemed to prefer dark oviposition areas adjacent to the illuminated sites, after it was initially attracted to the light. This could be because *S. latifolia* was not present in the dark areas adjacent to our illuminated sites, and therefore oviposition in the illuminated sites was the only option for *H. bicruris.* Interestingly, there was no difference between red light and the dark control suggesting that *H. bicruris* is not attracted by red light. The effect of light on fruit predation but not on fruit set may be due to differential responses of males and females to light, or of other species than *H. bicruris* pollinating *S. latifolia*, respectively. Whereas females both pollinate and oviposit in part of the pollinated flowers, males and other species only pollinate. Thus, if males or other species are less responsive to light or reduce their pollination activity due to light, fruit set will be less affected by light than fruit predation. The same would be true if females do not respond to light in terms of visitation and pollination, but are responsive to light in terms of oviposition decisions. Which of these two hypotheses is likely should be further investigated.

The results that the proportion of fertilized ovules (seed set) is lower under green light indicates that pollination visits under green light might be less efficient, interfering on another level with the plant–pollinator interaction. This analysis might be influenced by the higher infestation rate of *H. bicruris* under green light conditions. *H. bicruris* tends to oviposit eggs on the bigger fruits on primary branches^28^, which generally produce more seeds. Since we sampled fruits that were not infested we would have a bias for smaller fruits under treatments with a high infestation rate. Possibly these smaller fruits have a lower proportion of ovules setting seed. We indeed find a positive relationship between the total number of ovules and the ovule:seed ratio (F_1,268_ = 7.76, p < 0.01; Fig. S4). However, we do not find a lower total number of ovules for the fruits sampled under the green treatment (F_3,266_ = 2.37, p = 0.07; Fig. S5), which would be the case if there was a sampling bias. We therefore argue that this potential bias has very limited effects on our results.

Calculations of the number of seeds produced per flower indicate the potential impact of different light colours on plant reproduction. Despite the significant effects of light colour on infestation rates and ovule fertilization, the seeds produced per flower did not differ significantly between the four treatments. Considering the small sample size, the overall pattern indicates a potentially lower reproductive output under green light especially.

Even though most insects are fatally attracted to light and interactions disrupted (62% less interactions in Knop et al*.*^8^), there are insects which can cope with light at night. Here we worked with such a highly specialized system of which the main pollinator can cope with light, as shown experimentally in Giavi et al*.*^21^ and as can be concluded from the fact that *H. bicruris* also occurs in cities. In our case the shift in interactions due to light at night is a shift in the mutualism-antagonsim-continuum. It remains an open question how this will affect the long-term stability of the *Silene-Hadena* nursery pollination system. A key result is that light colour matters, with green and white light having the strongest effect and red light the least albeit that the number of seeds produced per flower did not differ with treatment. Therefore, red light is the light colour with the least impact on this plant–insect interaction. Our results thus highlight the possibility to reduce the impact of artificial light at night on ecological processes.

## Supplementary information

Supplementary Information.
