# Supplementary material for: Pollination and fruit infestation under artificial light at night:light colour matters
Source: Sci Rep. 2020 Oct 27;10:18389. doi: 10.1038/s41598-020-75471-1 (PMC7591485; doi:10.1038/s41598-020-75471-1)
Supplement: Supplementary file 1 — Supplementary Information. [file 41598_2020_75471_MOESM1_ESM.docx]

**SUPPLEMENATAL MATERIALS FOR:**

**Pollination and fruit infestation under artificial light at night: light colour matters**

**Michiel P. Boom^1^, Kamiel Spoelstra^1^, Arjen Biere^2^, Eva Knop^3,4^ & Marcel E. Visser^1*^**

^1^Department of Animal Ecology, Netherlands Institute of Ecology (NIOO-KNAW), P.O. Box 50, 6700 AB, Wageningen, The Netherlands

^2^ Department of Terrestrial Ecology, Netherlands Institute of Ecology (NIOO-KNAW), P.O. Box 50, 6700 AB, Wageningen, The Netherlands

^3^ University of Zürich, Departement of Evoluationary Biology and Environmental Studies, Winterthurerstr. 190, 8057 Zürich, Switzerland

^4^ Agroscope, Agroecology and Environment, Reckenholzstr. 191, 8046 Zürich, Switzerland

*Corresponding author: m.visser@nioo.knaw.nl

*Running title:* Effects of light on pollination

**Supplementary figure 1**

*Figure S1. Spectral composition of the green (Philips Fortimo Clearsky; green line), red (Philips Fortimo ClearField; red line) and white light (Philips Fortimo White; yellow line), from Spoelstra et al.*^1^.

**Supplementary figure 2**

**
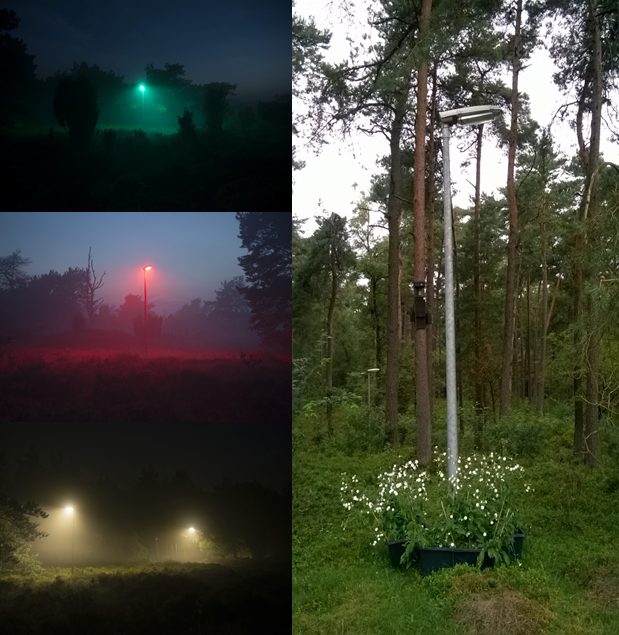
**

*Figure S2: Impression of the experimental set up with images of lampposts of the green, red and white transect (left, photos by Kamiel Spoelstra); and an image of plants of S. latifolia directly underneath a lamppost (right, photo by Michiel P. Boom).*

**Supplementary figure 3**


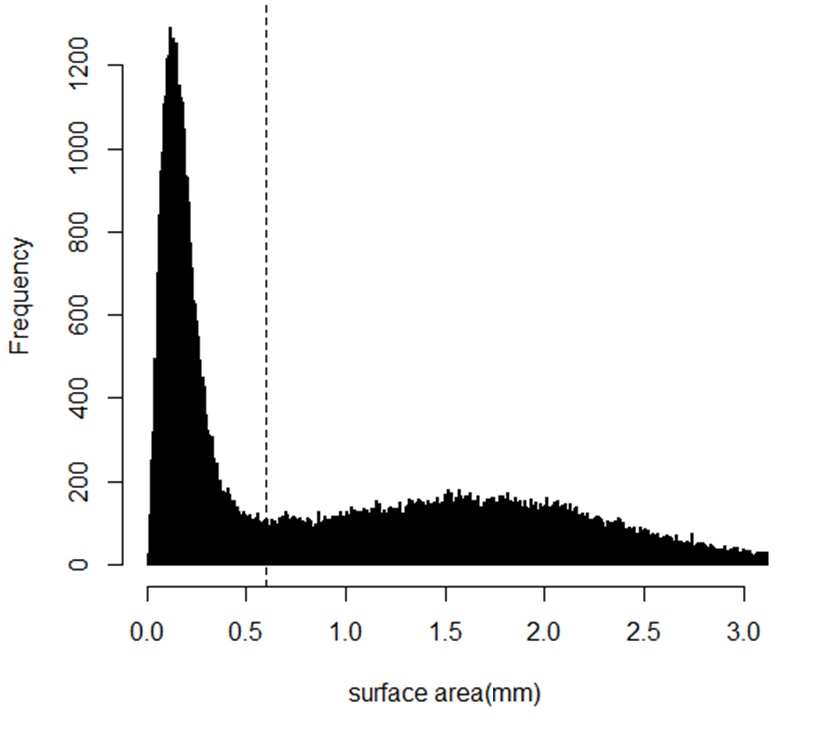


*Figure S3: Histogram of seed surface area used to distinguish between fertilized and unfertilized seeds. The dashed line indicates the threshold used at 0.6 mm^2^*

**Supplementary figure 4**
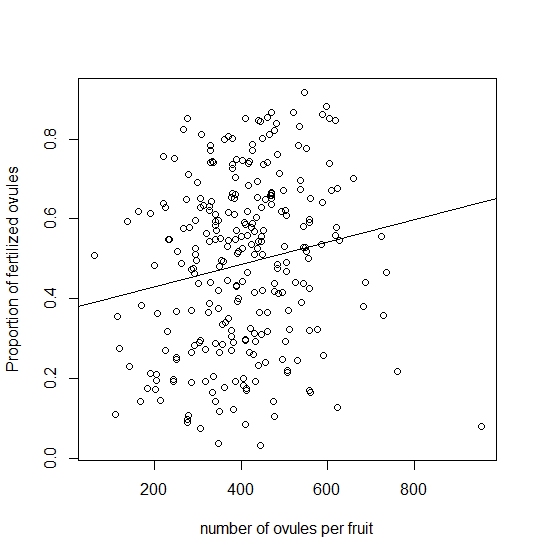
 *Figure S4: Relationship between the number of ovules per fruit and the proportion of fertilized ovules. Estimate = 0.00028 ± 0.0001,* F_1,268_ = 7.76, p < 0.01.

**Supplementary figure 5**


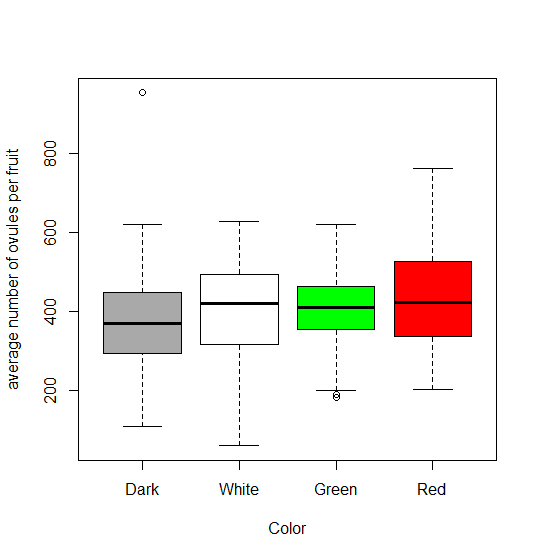


*Figure S5: The average number of ovules per fruit for the four different light treatments. Differences between treatments were not significant (*F_3,266_ = 2.37, p = 0.07*).*

**References**

1. Spoelstra, K. *et al.* Experimental illumination of natural habitat—an experimental set-up to assess the direct and indirect ecological consequences of artificial light of different spectral composition. *Phil. Trans. R. Soc. B* **370**, 20140129 (2015).
